# How Should We Care for Patients with Congenital Heart Diseases Undergoing Surgical Procedures in Ambulatory Settings?

**Published:** 2022

**Authors:** Rebecca Hamilton, Kirsten C Odegard, Koichi Yuki

**Affiliations:** Cardiac Anesthesia Division, Department of Anesthesiology, Critical Care and Pain Medicine, Boston Children’s Hospital, USA

## Abstract

Congenital heart diseases (CHDs) are the most common of all congenital birth anomalies. As the survival of patients with CHDs continues to improve, this patient population is presenting for non-cardiac procedures more frequently than in the past. With ambulatory based procedures becoming increasingly common, it is critical to consider how we should best triage these patients for procedures in ambulatory settings. This paper reviews the current literature on the subject and considers strategies to guide future management.

## Introduction

The introduction of the first freestanding ambulatory surgery centers (ASCs) in 1970s resulted in a rapid increase in outpatient-based surgery [1,2]. Improvements in surgical and anesthetic techniques have increased the proportion of outpatient-based surgery to > 70% of the total surgical procedures in the U.S [3]. One of the most important criteria for successful conduction of outpatient-based procedures is minimizing unplanned hospital admission, and the selection of type of procedures and patient cohort would be a key.

Congenital heart disease (CHD), defined as a gross structural anomaly of the heart or intrathoracic great vessels that is actually or possibly of functional significance, is the most common group of live birth anomalies with a prevalence of 4–10/1,000 live births [4]. Although a number of CHD lesions were previously fatal, over 90% of children now survive to adulthood due to medical improvement [5]. As a result, the number of adults has exceeded children living with CHD in high-income countries, and the number of patients with CHD is expected to keep growing by approximately 5% per year [6]. Considering these statistics, it is natural to expect that an increasing number of patients with CHD will seek for medical attention due to issues not directly associated with their cardiac conditions, some of which involve a wide range of procedures. As CHD is considered a risk factor for perioperative complications, and outpatient-based procedures are becoming increasingly popular, it comes as no surprise that there is a great interest in understanding the feasibility of outpatient based procedures in this cohort. In this paper, we will review the current knowledge on risk factors of perioperative complications in patients with CHD and pediatric ambulatory procedures.

## Outcomes and Risk Factors of Non-Cardiac Surgery in Patients with Congenital Heart Disease

The outcomes of non-cardiac surgeries in patients with CHD have been studied by a number of investigators. Baum, et al. did a retrospective data analysis of hospitalized children using multi-institutional database from 1993 to 1996 [7]. In this cohort, 6.5% of 191,261 patients < 18-years-old had been diagnosed with CHD. Children with CHD had a 30-day mortality rate of 6%, compared to 3.8% in patients without CHD. Of all CHDs, hypoplastic left heart syndrome (HLHS) is considered one of the most challenging to manage in the perioperative context. Torres, et al. used the nationwide inpatient sample covering 1,000 hospitals in 22 states in the US [8]. 2,457 children aged less than 2 years with the diagnosis of HLHS were identified to have non-cardiac surgery from 1988 to 1997. The mortality rate in this selected cohort was 19%. Certainly, these data were enough to alarm healthcare providers that CHDs posed a significant risk for perioperative mortality. At the same time, an improvement of CHD management, surgery and anesthesia has continued to contribute to the enhancement of outcomes in this cohort in general. Therefore, the data from two decades ago may not necessarily reflect current situation of non-cardiac surgeries in this population. For example, Hennein, et al. examined the risk factors for mortality in 208 patients with CHD who underwent a total of 228 general surgery procedures under general anesthesia from 1981 to 1991. Identified risk factors for perioperative mortalityin this cohort included an American Society of Anesthesia (ASA) class IV, preoperative hospital length of stay > 10 days, birth at a tertiary care center, and emergency operations. However, this study was conducted at a time when outpatient procedures were first being introduced into the pediatric arena, and these risk factors may no longer be as applicable to our practice today.

One of difficulties in caring for patients with CHDs is the wide range of diversity with which these lesions present. In an attempt to classify severity, CHDs are commonly divided into three subtypes; Minor, major and severe CHDs [9]. Minor CHD is defined as a cardiac condition with or without cardiac-related medications and maintenance, or post repair of congenital heart defect with normal cardiovascular function and no medications (Table 1). Examples of minor CHDs include patients with atrial septal defect (ASD) or with small-to-moderate sized ventricular septal defect (VSD). Major CHD is defined as a CHD post-repair with residual hemodynamic abnormality with or without medications. Examples of major CHDs include patients who have undergone Tetralogy of Fallot (TOF) repair with wide open pulmonary insufficiency or Stage 1 repair for HLHS. Severe CHD is typically reserved for patients with uncorrected cyanotic heart disease, any documented pulmonary hypertension, ventricular dysfunction requiring medications, or listed for heart transplant. In 2012, Faraoni, et al. analyzed the pediatric database of the American College of Surgeons National Surgical Quality Improvement Program (ACS NSQIP) using this classification to examine postoperative outcomes in children with and without minor, major and severe CHDs undergoing non-cardiac surgery [9]. The 30-day mortality of patients with minor, major and severe CHDs was 1.1%, 2.2% and 5.7%, respectively. There was no difference in the outcomes between patients without CHD and patients with minor CHD. The results suggested that while the classification of minor, major and severe CHDswas practical for providers, there was a gap in the understanding of what additional risk factors were associated with the outcome of non-cardiac procedures in this patient population. A decade earlier, Warner, et al. had performed a retrospective review of all patients with CHDs < 50-years-old who underwent non-cardiac surgery at Mayo affiliated hospitals between 1987 and 1992 [10]. They identified 276 patients who underwent 480 non-cardiac procedures. The primary outcome was perioperative complications, defined as the onset of a new cardiac problem or exacerbation of a previously stable cardiac condition, onset of a new respiratory problem or exacerbation of a previously stable respiratory condition, and onset of a new neurological deficit. Risk factors associated with complications were age < 2 years, cyanosis, pulmonary hypertension, active treatment for congestive heart failure, and poor general health at the time of the procedures. Other than age, the rest of risk factors were very similar to the criteria for severe CHD in ACS NSQIP, which supports the use of the minor, major and severe CHD classification.

Faraoni, et al. went on to examine the 2012–2014 ACS NSQIP database and compared 4,375 major and severe CHD with a validation cohort. The mortality of patients with major and severe CHD who underwent noncardiac procedures was 4.7%. Mortality risk factors included emergency procedure, severe CHD, single-ventricle physiology, previous surgery within 30 days, preoperative inotropic support, preoperative CPR, acute/chronic renal injury and preoperative mechanical ventilation. Then, how about intraoperative events? For ambulatory procedures, understanding the type and risk factors for intraoperative events would be important. Lee, et al. examined a single institutional data from 2008 to 2013 in patients who underwent noncardiac procedures in patients with CHD [11], capturing cardiovascular events as well as respiratory events. Cardiovascular evens included continuous inotrope infusion administration, arrhythmias requiring treatment, pulmonary hypertensive crisis, and cardiac arrest. Respiratory events included bronchospasm/laryngospasm, difficult intubation, failed extubation/reintubation, and prolonged desaturation. ASA class III or above, major and severe CHDs, single ventricle physiology and mild-severe ventricular dysfunction were risk factors for cardiovascular events. Respiratory events were associated with only ASA III class or above. Together, these results support screening patients with CHD scheduled for ambulatory procedures for major and severe CHD, single ventricle physiology, ventricular dysfunction, and high ASA class to identify patients at most risk for complications.

Because CHDs contain diverse diseases, identifying risk factors for perioperative complications would be useful as we discussed above. However, we also need to keep in mind to anesthetize those patients tailored for individual circulatory patterns. For example, Fontan circulation, one of well-known single ventricular circulation, has very small unstressed volume [12]. Administration of anesthesia and resultant vasodilation can cause significant volume shift from stressed volume to unstressed volume in this physiology, often requiring volume bolus and/or administering vasoactive drugs [12]. Thus, familiarization to each physiology is critical even after screening in CHD patients for ambulatory procedures.

## Data of Ambulatory Surgery in Patients with or without Congenital Heart Disease

As described above, an important quality and safety metric in the evaluation of ambulatory surgery programs is the incidence of unplanned hospital admission. In adults, the incidence of unplanned hospital admission is 2.7% [13]. In the pediatric population, the incidence of unplanned hospital admissions is generally lower. Awad, et al. retrospectively analyzed 10,772 children who underwent ambulatory surgery from January 1996 to December 1999 in an academic tertiary children’s hospital in Ireland [14] (Table 2). The incidence of unplanned hospital admission was 2.2% (242 cases). The most common reason for admission was issues related to surgery (54%); other reasons included anesthesia-related issues (postoperative nausea and vomiting, somnolence) (16%), social issue due to later surgery (surgery ends after 3 pm) (14%) and medical issues (11%). Similarly, Rabbitts, et al. compared all pediatric ambulatory procedures conducted in the US in the 1996 and 2006 National Survey of Ambulatory Surgery (NSAS) [3]. In 1996, ambulatory anesthesia was provided in 1,522,833 cases for patients younger than 15 years. In 2006, ambulatory anesthesia was provided in 2,300,651 cases, supporting the notion that pediatric ambulatory anesthesia has increased over time just as it has in the adult cohort. In 2006, 0.6% of cases required admission. Whippey, et al. examined ambulatory surgery in children < 18 years (and > 60 weeks post conceptual age) in a single pediatric tertiary hospital in Canada from 2005 to 2012. Out of 21,957 cases, 213 cases (0.97%) required admission. The risk factors for admission were ages < 2 years, ASA class III, duration of surgery > 1-hour, surgical end time later than 3 pm, type of surgery (specifically orthopedics, otolaryngology, dental), intraoperative events and the presence of obstructive sleep apnea [15]. Intraoperative events included difficult intubation, bronchospasm/laryngospasm, aspiration, blood loss requiring transfusion and cardiac arrest.

Then, how about patients with CHD? Yuki, et al. performed retrospective analysis of the data from a single pediatric institution in the US from 2008 to 2013 [16]. Out of 3,010 non-cardiac procedures in patients with CHD, 1,028 cases (34.1%) were scheduled to have ambulatory procedures. The rate of unplanned admission was 2.8%. Reasons for admission were mostly related to anesthesia including postoperative nausea and vomiting, hypoxemia or intubation pain, fever, headache, and agitation. The risk factors for admission were ASA class III or higher, major CHD, the availability of an echocardiogram within 6 months of procedures, and radiology procedures. Authors speculated that an echocardiogram was likely obtained frequently in patients with CHD whose residual lesions or ventricular function were concerning enough to be followed closely. Patients who underwent procedures in radiology were younger, of higher ASA classification status and presented more frequently with major CHD and associated syndromes in this study.

## Current Non-Cardiac Surgery in CHD Cohort

As a recent survey of non-cardiac surgical volume in US pediatric centers demonstrated, ambulatory procedures are increasing in volume, from 19,719 cases in 2015 to 25,890 cases in 2019 [17]. Although the incidence of admission was not reported in this study, the mortality (0.02%) in ambulatory procedures was significantly lower compared to that of inpatients (3.07%), which supports performing non-cardiac procedures in the ambulatory setting even for the CHD cohort. In reality, however, when we talk about ambulatory surgery, we need to differentiate the setup where ambulatory surgery is performed. Ambulatory surgery can be done in a freestanding ambulatory surgery center with limited resources, a satellite hospital affiliated witha main pediatric institution but primarily designed for outpatient care, and a large pediatric institution with inpatient care capacity. Care at a large pediatric institution with inpatient care capacity requires more resources compared to care received at other settings. Additionally, one could speculate that the screening process for ambulatory surgery at a large pediatric center might be less strict compared to satellite locations. Although the literature on screening processes at such locations is limited, this should be considered in depth. Maxwell, et al. used a comprehensive state ambulatory surgical registry (the California Ambulatory Surgery Database, 2005–2011) to examine where pediatric patients which CHD were cared for when undergoing nonsurgical procedures [18]. Among 11,254 cases, 43% of cases were cared at non-CHD specialty centers. 29% of cases were done at the nearest CHD center, and 28% of cases were done at other CHD centers. Patients who underwent procedures at non-CHD specialty centers lived farther from the nearest CHD center than patients who underwent care at a designated CHD center, indicating that access to care and systems structures may factor into decisions made regarding ambulatory procedures in this patient cohort. If issues were to arise at a non-CHD specialty center, would these patients be transferred to a CHD center, potentially skewing the data? Screening criteria for ambulatory surgery in CHD patients should be individualized to the capabilities of each healthcare setting and include a plan should postoperative admission requirements arise unexpectedly. The American College of Cardiology/American Heart Association consensus guidelines recommend that adult patients with CHD receive perioperative care in specialized adult CHD centers [19]. However, in reality more than half of adult patients with CHD are cared for at non-specialty center [18], which may be reflective of a growing adult CHD population as well as limitation of available healthcare resources and their uneven geographical distribution. Considering that the need for non-cardiac surgeries is increasing in the pediatric CHD population, it is critical to employ a society-wide multi-disciplinary approach when determining how these patients should be cared for, particularly in the context of ambulatory procedures. This consideration should be applicable for both pediatric and adult CHD patients.

## Future Direction

As suggested in the literature reviewed here, ambulatory procedures in the CHD population are on the rise. However, there is no strong evidence on how to determine patient selection for procedures in ambulatory settings. As risk factors for ambulatory procedures continue to be evaluated, the establishment of a triage guideline for ambulatory procedure feasibility would be of tremendous importance. Another missing piece is the information of how adult CHDs should be cared for. As this population grows, we need to understand the care for adult CHDs in this setting. Further research is needed to sophisticate and streamline the provision of surgical care for patients with CHD in ambulatory settings.

## Figures and Tables

**Table 1:** CHD classification.

Minor CHD	Cardiac condition with or without medication and maintenance Repair of CHD with normal cardiovascular function and no medication
Major CHD	Repair of CHD with residual hemodynamic abnormality with or without medications
Severe CHD	Uncorrected cyanotic heart disease Patients with any documented pulmonary hypertension Patients with ventricular dysfunction requiring medications Listed for heart transplant

**Table 2:** Unplanned admission following planned ambulatory procedures.

Study	Incidence of unplanned admission	Risk factors of unplanned admission
Awad, et al. [14]	2.2%	Issues related to surgery, anesthesia related issues (PONV, somnolence), late surgery end, medical issues
Whippey, et al. [13]	0.97%	< 2 years, ASA class III~, surgery > 1 hour, surgical end time > 3 pm, orthopedics/otolaryngology/dental surgery, intraoperative events (Difficult intubation, bronchospasm/laryngospasm, aspiration, blood transfusion, cardiac arrest), OSA
Yuki, et al. [16]	2.8%	ASA III~, major CHD, radiology procedure, recent echo availability

PONV: Postoperative Nausea and Vomiting; OSA: Obstructive Sleep Apnea
